# Myositis, Vasculitis, Hepatic Dysfunction in Adult-Onset Still's Disease

**DOI:** 10.1155/2009/504897

**Published:** 2009-06-21

**Authors:** Hidekatsu Yanai, Nobuyuki Furutani, Hiroshi Yoshida, Norio Tada

**Affiliations:** ^1^Division of General Medicine, Department of Internal Medicine, The Jikei University School of Medicine, 163-1, Kashiwashita, Kashiwa, Chiba 277-8567, Japan; ^2^Department of Laboratory Medicine, The Jikei University School of Medicine, 163-1, Kashiwashita, Kashiwa, Chiba 277-8567, Japan

## Abstract

Although hepatic dysfunction is common in adult-onset Still's disease (AOSD), sometimes it is difficult to differentiate hepatic dysfunction due to AOSD itself from drug-induced hepatic dysfunction. Further, myalgia often occurs in patients with AOSD; however, AOSD patients complicated with myositis are rare. We report a 43-year-old Japanese man with AOSD who developed myositis and hepatic dysfunction which were deteriorated by multiple nonsteroidal antiinflammatory drugs (NSAIDs) and were dramatically ameliorated by a low-dose steroid therapy. A skin biopsy of salmon pink rash which is characteristic for AOSD showed leukocytoclastic vasculitis, and the markers for vasculitis, plasma von Willebrand factor, and vascular endothelial growth factor levels were elevated in this patient, suggesting an association between AOSD and systemic vasculitis.

## 1. Introduction

Adult-onset Still's disease (AOSD) is a rare systemic inflammatory disease of unknown etiology. Hepatic dysfunction is common in AOSD, and more than 70% of these patients show elevated liver enzymes [1]. Evanescent salmon-pink rash is characteristic for AOSD; however, the histopathology of this rash has not been cleared. Although myalgia often occurs in AOSD, myositis complicated with AOSD is rare [2]. Here, we describe a patient with AOSD who developed myositis and hepatic dysfunction which were deteriorated by multiple nonsteroidal anti-inflammatory drugs (NSAIDs) and were dramatically ameliorated by a low-dose steroid therapy. Further, we also show the association between AOSD and vasculitis by presenting the pathologic findings of salmon-pink rash and elevated markers for vasculitis in AOSD.

## 2. Case Presentation

**Figure 1:**
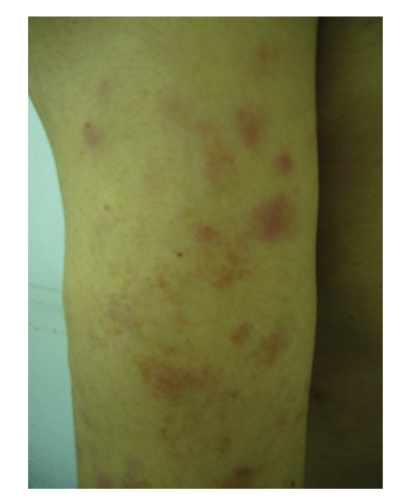
Salmon-pink rash on the patient's upper arm.

A 43-year old Japanese man was referred and admitted with a continuous spiking fever, myalgia, and arthralgia. Physical examination revealed fever, muscle weakness, and salmon-pink rash (Figure 1). Laboratory examination was notable for a C-reactive protein (CRP) level of 1.1 mg/dL (normal range, 0.0–0.3), a fibrinogen level of 504 mg/dL (normal range, 150–400), a creatin kinase (CK) level of 1.114 IU/L (normal range, 55–200), and a ferritin level of 2.892 ng/mL (normal range, 40–350). Serum rheumatoid factor, antinuclear antigen, proteinase 3 (PR3)- and myeloperoxidase (MPO)-antineutrophil cytoplasmic autoantibodies (ANCA), and anti-Jo-1 antibody were negative. He was diagnosed as AOSD based on Yamaguchi's criteria [3].

**Figure 2:**
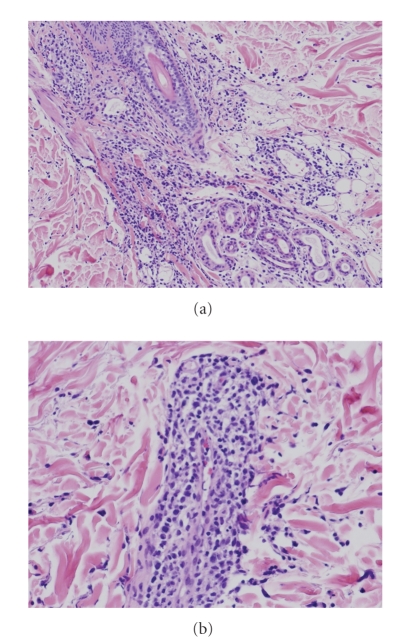
Histopathological findings from a salmon-pink rash showed perivascular lymphocytes infiltration and fragmentation of blood cells, which was compatible with leukocytoclastic vasculitis. (Hematoxylin-eosin stain; original magnification: ×25 (a) and ×50 (b).)

Needle electromyography showed active myogenic change in right biceps femoris muscle and deltoid muscle. A skin biopsy of salmon pink rash showed perivascular lymphocytes infiltration and fragmentation of blood cells, which was compatible with leukocytoclastic vasculitis (Figure 2). Markers for vasculitis, plasma von Willebrand factor (vWF) (235% (normal range, 60–170)), and vascular endothelial growth factor (VEGF) (1.050 pg per milliliter (normal range <115)) were elevated.

**Figure 3:**
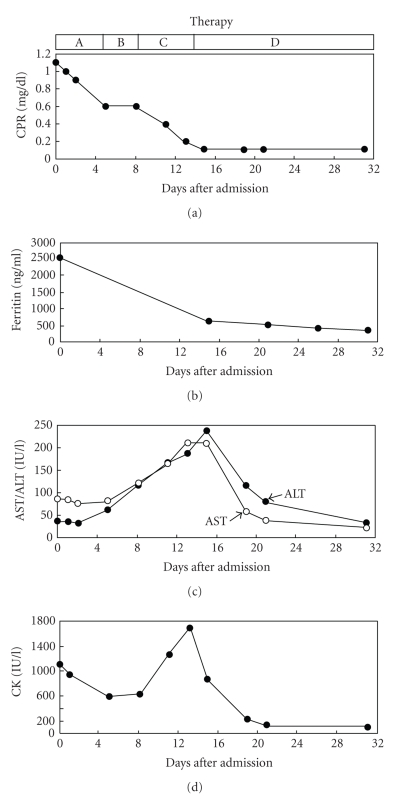
Changes in serum C-reactive protein (CRP), ferritin, aspartate aminotransferase (AST), alanine aminotransferase (ALT), and creatin kinase (CK) levels in a case with adult-onset Still's disease. Therapies A, B, C, and D mean acetaminophen (1500 mg/d), loxoprofen sodium (180 mg/d), meloxicam (10 mg/d), and corticosteroid (20 mg/d), respectively.

Acetaminophne, loxoprofen sodium, and meloxicam decreased his fever, serum CRP, and ferritin levels; however, these NSAIDs elevated serum aspartate aminotransferase (AST), alanine aminotransferase (ALT), and CK levels (Figure 3). A 20 mg corticosteroid therapy promptly ameliorated patient's symptoms and decreased serum AST, ALT, and CK (Figure 3).

## 3. Discussion

To our knowledge, the histopathology of evanescent salmon-pink rash has not been cleared. A skin biopsy revealed that rash was due to leukocytoclastic vasculitis. Leukocytoclastic vasculitis is characterized by angiocentric segmental inflammation, fibrinoid necrosis, and a neutrophilic infiltrate around the vessel walls with erythrocyte extravasation [4]. Leukocytoclastic vasculitis has been observed in Henoch-Schönlein purpura, Wegener's granulomatosis, and microscopic polyangiitis [5–7]; however, it has not been reported in AOSD. Negative PR3- and MPO-ANCA denied complication with Wegener's granulomatosis or microscopic polyangiitis in our patient.

vWF is produced by blood vessel endothelial cells in response to injury, and high vWF level has been observed in systemic vasculitis [8]. VEGF is a cytokine participating in inflammation with potent endothelial effect and is produced by macrophages, neutrophils, and vascular endothelial cells and can alter vascular permeability. VEGF has also been suggested to be associated with vasculitis, and elevated serum VEGF levels have been observed in Behçet's disease, microscopic polyangiitis, polyarteritis nodosa, and giant cell arteritis [9–12]. Elevated blood vWF and VEGF levels in our AOSD patient suggest a significant association between AOSD and vasculitis. We have to mention that VEGF immunostaining on the cutaneous lesion would be more relevant than serum VEGF measurement, and that we did not study serum vWF and VEGF levels after the therapy; however, investigation of changes in their levels after the corticosteroid therapy would be helpful to show their role in the pathogenesis of vasculitis in this patient.

Liver dysfunction in AOSD has been ever described in some case reports. Andrès et al. retrospectively reviewed data of 17 patients with AOSD and found abnormalities in liver biochemistry in 76% of subjects with AOSD, suggesting the high frequency of liver dysfunction in AOSD [1]. However, it is difficult to differentiate liver dysfunction due to AOSD itself from drug-induced liver dysfunction. Recently, Chen et al. determined soluble intercellular adhesion molecule 1 (sICAM-1) in patients with active untreated AOSD and identified serum sICAM-1 level as a predictor of liver dysfunction in AOSD, and serum sICAM-1 levels were significantly correlated with disease activity and serum ferritin levels which have been also reported to be suitable for monitoring AOSD disease activity [13]. In our subject, in spite of decrease in serum CRP and ferritin levels, serum AST and ALT levels were increased by NSAIDs and were promptly decreased by corticosteroid, suggesting the presence of drug-induced liver dysfunction. In this case, NSAIDs also elevated serum CK levels, and a 20 mg corticosteroid therapy promptly decreased serum CK levels, suggesting NSAIDs-induced myositis. To our knowledge, drug-induced myositis in AOSD has not been previously reported. Schwarz-Eywill et al. have also observed that aspirin improved symptoms and CRP but increased serum AST and ALT levels in AOSD patients, and a corticosteroid treatment decreased serum AST and ALT levels [14]. AOSD may have a potential mechanism for enhancing drug toxicity. If we find deterioration in liver function and myositis during treatment, in spite of decrease in CRP and ferritin, we should be aware of drug-induced hepatitis and myositis in AOSD.

In conclusion, our observation indicates that AOSD may be associated with systemic vasculitis and also suggests that we should be aware of the presence of drug-induced hepatitis and myositis in AOSD.
